# Supplementary figures and images for: Insights into substrate binding and utilization by hyaluronan synthase
Source: eLife. 2026 Mar 13;14:RP109624. doi: 10.7554/eLife.109624 (PMC12987647; doi:10.7554/eLife.109624)

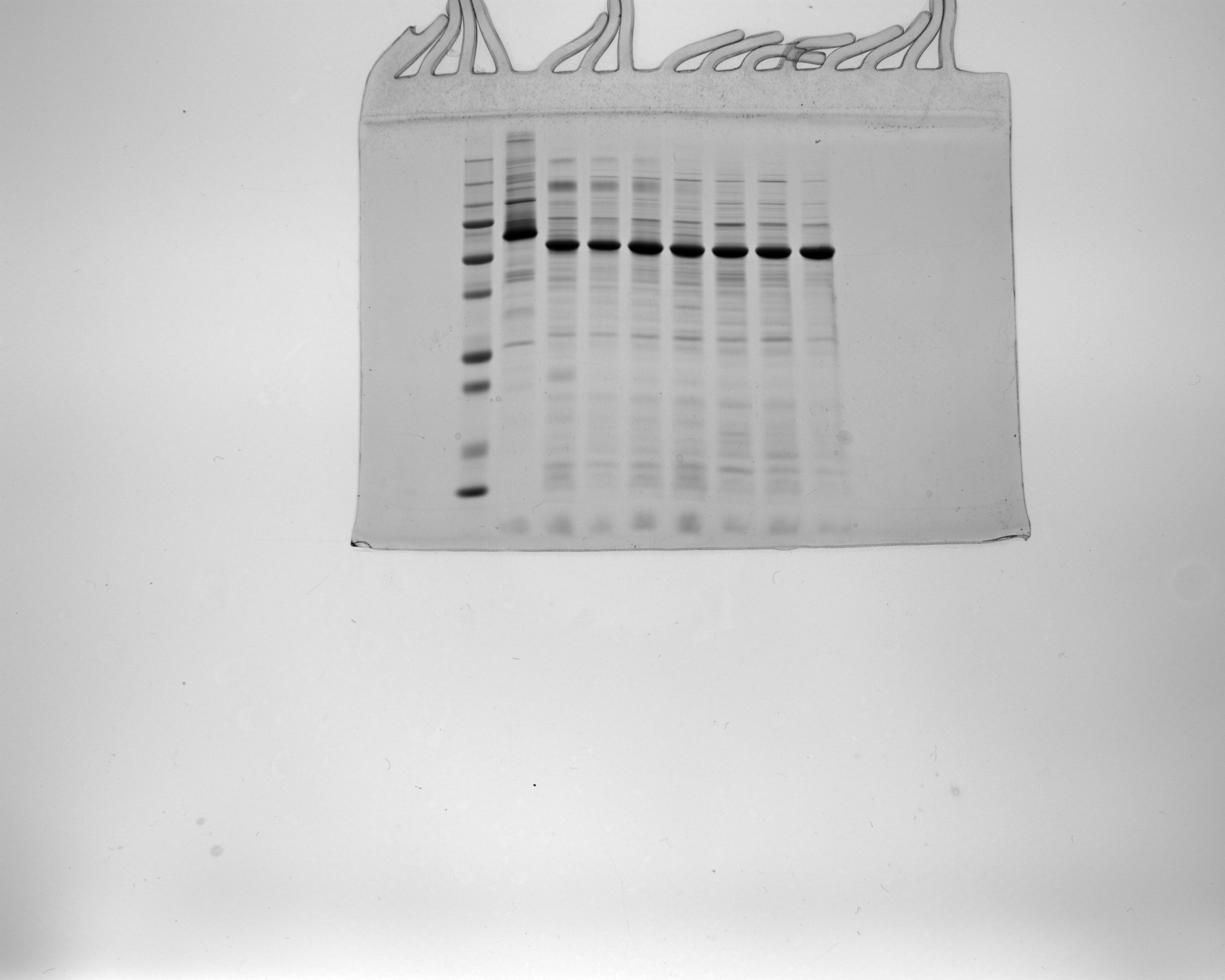

Supplement: Figure 1—figure supplement 3—source data 1. [file elife-109624-fig1-figsupp3-data1.zip › Figure 1 - figure supplement 3 - source data 1.tif]

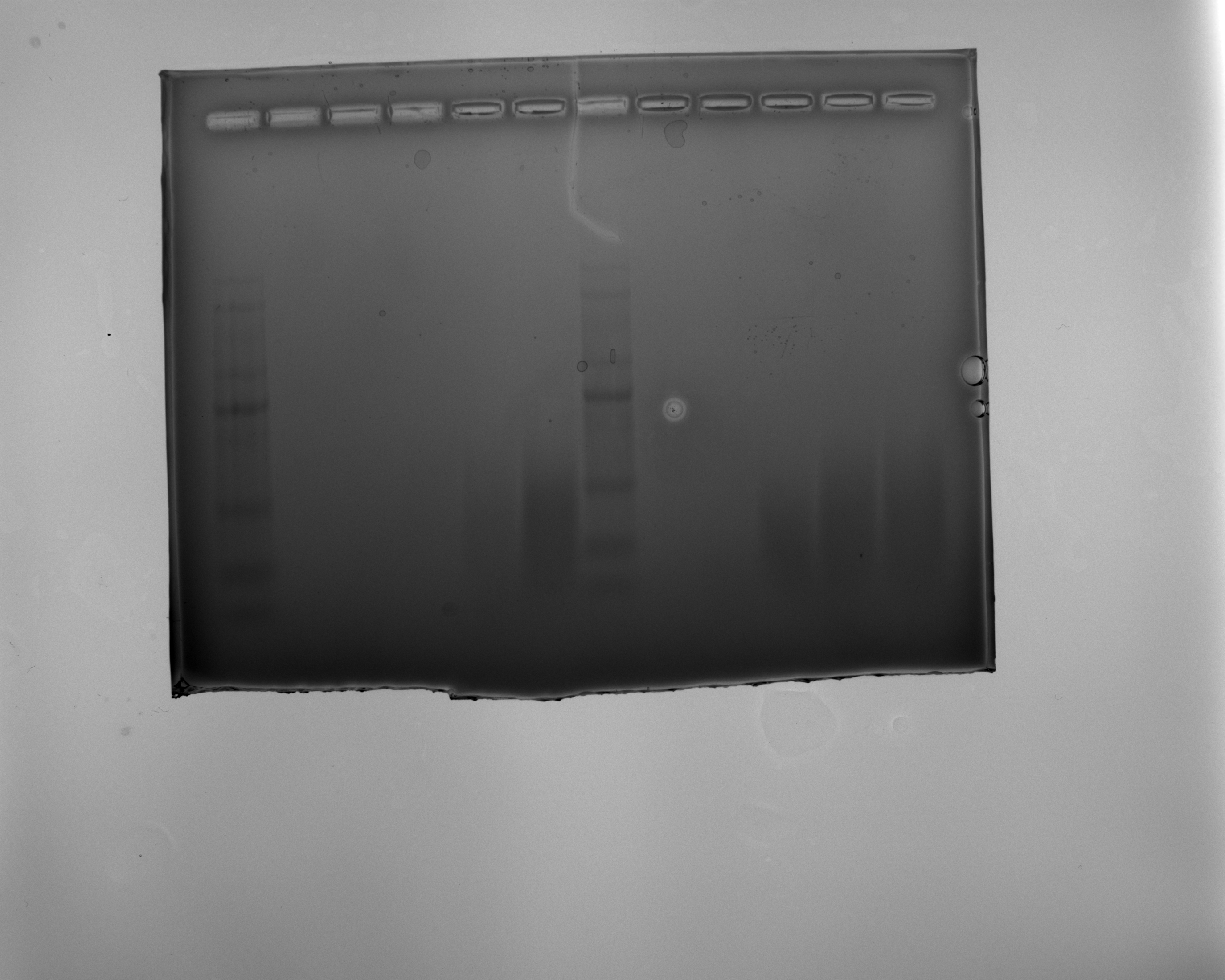

Supplement: Figure 2—source data 1. [file elife-109624-fig2-data1.zip › Figure 2 - source data 1.tif]

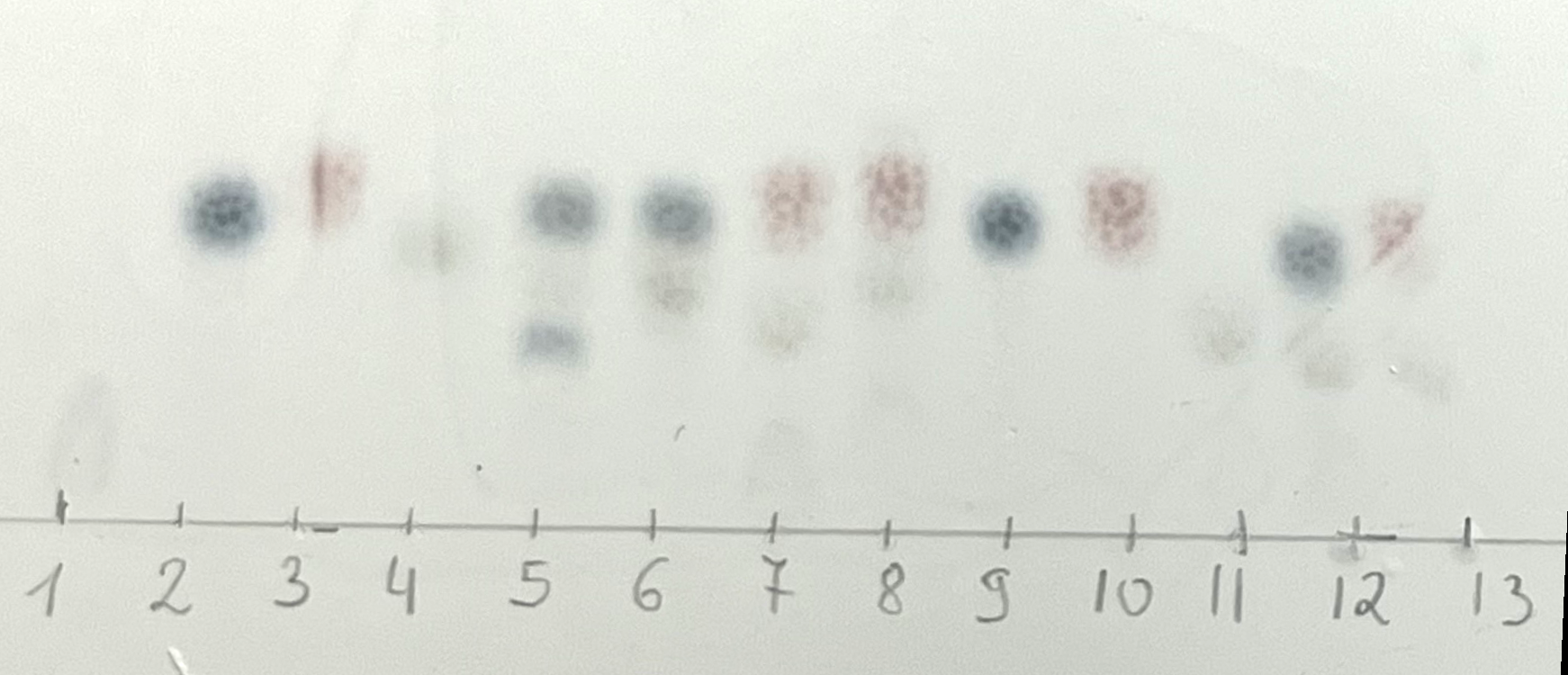

Supplement: Figure 4—source data 1. [file elife-109624-fig4-data1.zip › Figure 4 - source data 1 - 1.tif]

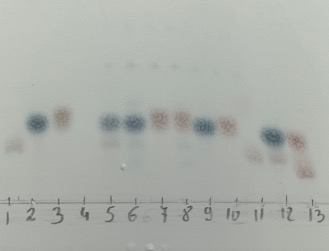

Supplement: Figure 4—source data 1. [file elife-109624-fig4-data1.zip › Figure 4 - source data 1 - 2.tif]

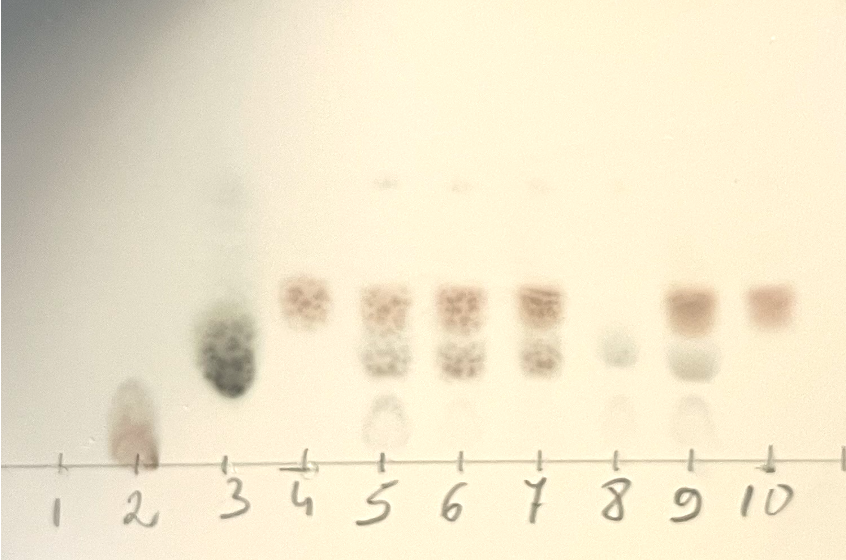

Supplement: Figure 4—figure supplement 2—source data 1. [file elife-109624-fig4-figsupp2-data1.zip › Figure 4 - figure supplement 2 - source data 1 - 2.tif]

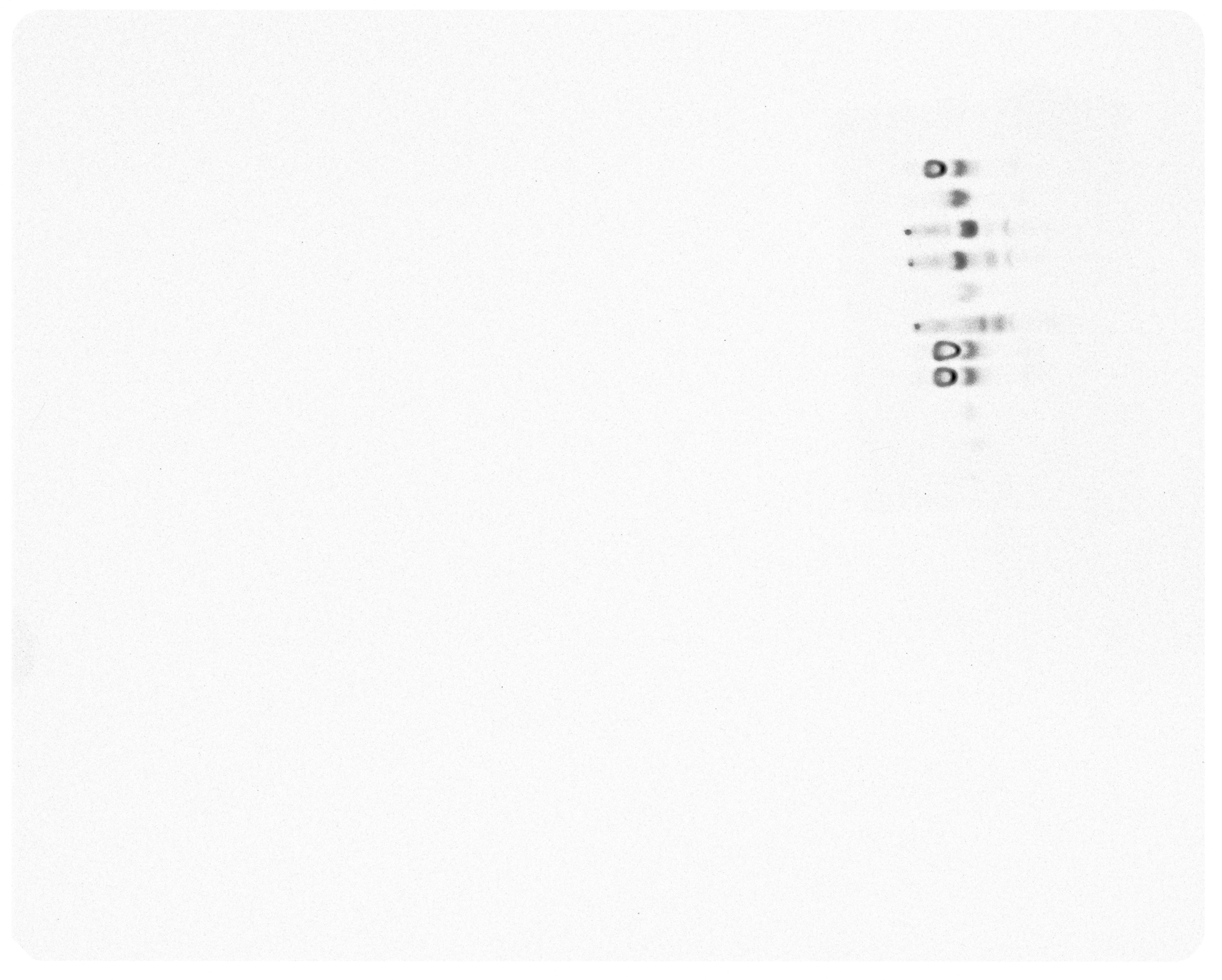

Supplement: Figure 4—figure supplement 2—source data 1. [file elife-109624-fig4-figsupp2-data1.zip › Figure 4 - figure supplement 2 - source data 1 - 3.tif]

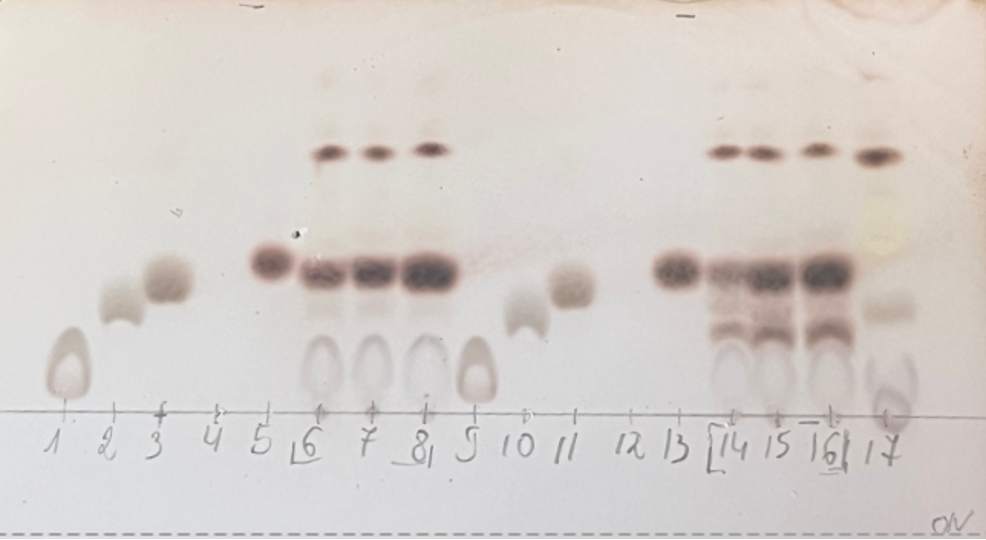

Supplement: Figure 4—figure supplement 2—source data 1. [file elife-109624-fig4-figsupp2-data1.zip › Figure 4 - figure supplement 2 - source data 1 - 1.tif]
