# Supplementary material for: Insights into substrate binding and utilization by hyaluronan synthase
Source: eLife. 2026 Mar 13;14:RP109624. doi: 10.7554/eLife.109624 (PMC12987647; doi:10.7554/eLife.109624)
Supplement: Figure 2—source data 2. [file elife-109624-fig2-data2.pdf]

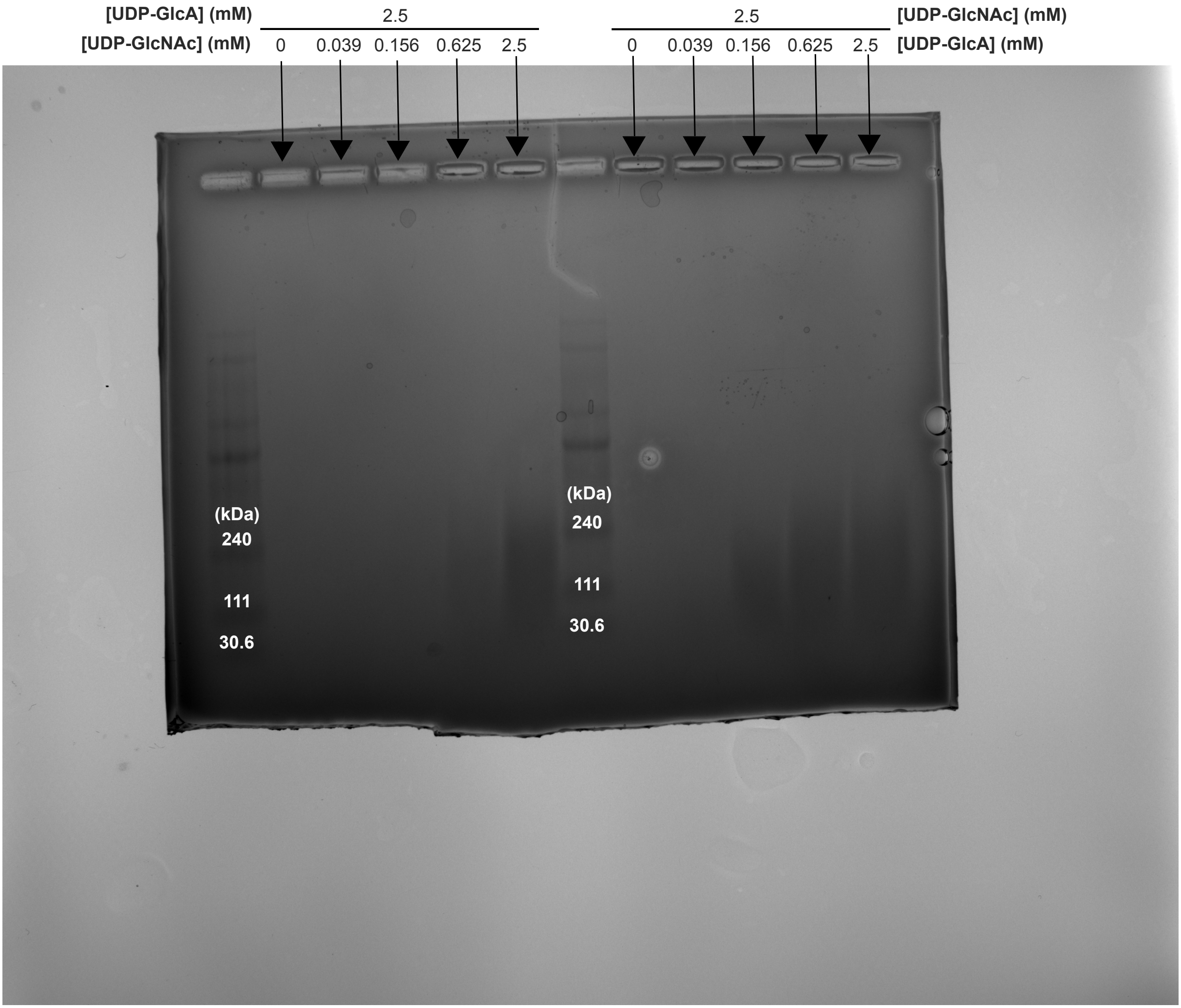

Figure 2 – source data 2: Stains-All gel of CvHAS HA synthesis products under substrate limiting conditions with relevant lanes labeled.
