# Supplementary material for: Insights into substrate binding and utilization by hyaluronan synthase
Source: eLife. 2026 Mar 13;14:RP109624. doi: 10.7554/eLife.109624 (PMC12987647; doi:10.7554/eLife.109624)
Supplement: Figure 4—source data 2. [file elife-109624-fig4-data2.pdf]

CvHAS	-	-	-	-	+	+	+	+	-	-
UDP-GlcA	+	-	-	-	+	-	+	-	+	+
Cellobiose	-	+	-	-	+	+	-	-	+	-
Chitobiose	-	-	+	-	-	-	+	+	-	+
GlcA	-	-	-	+	-	+	-	+	-	-
UDP	-	-	-	-	-	+	-	+	-	-

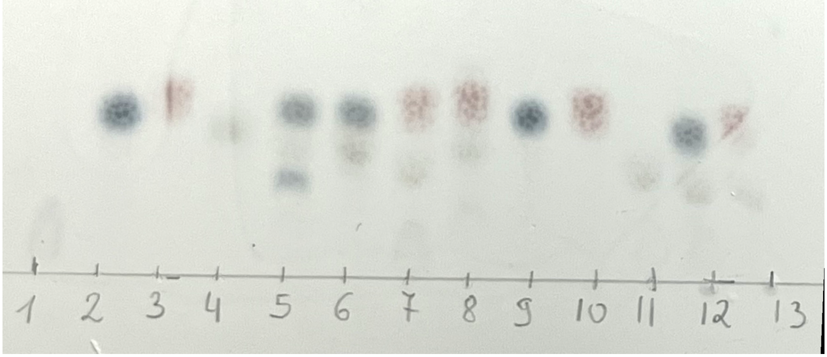

CvHAS	-	-	-	-	+	+	+	+	-	-
UDP-GlcNAc	+	-	-	-	+	-	+	-	+	+
Cellobiose	-	+	-	-	+	+	-	-	+	-
Chitobiose	-	-	+	-	-	-	+	+	-	+
GlcNAc	-	-	-	+	-	+	-	+	-	-
UDP	-	-	-	-	-	+	-	+	-	-

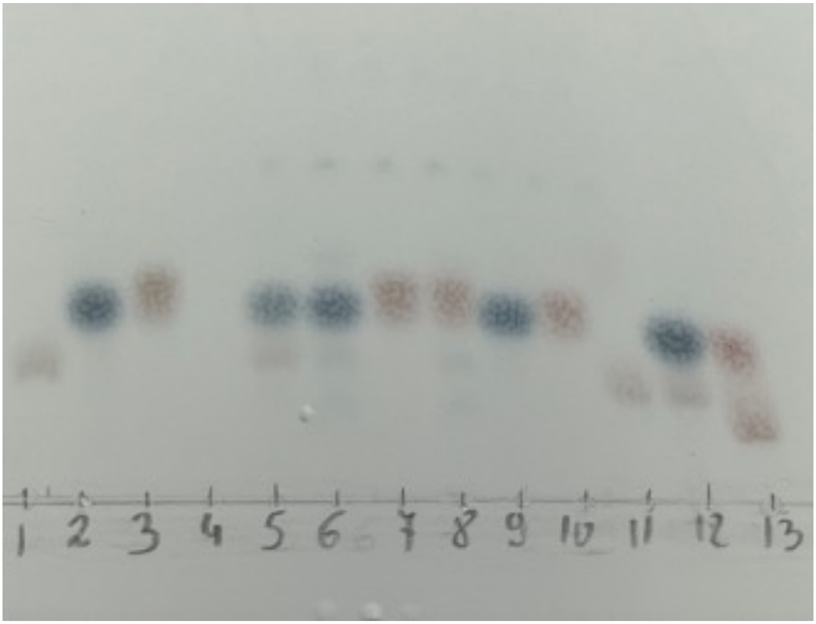

Figure 4 - source data 2: Diphenylamine exposed TLC plates displaying putative cellobiose and chitobiose primer extension by GlcA.
