# Supplementary material for: Insights into substrate binding and utilization by hyaluronan synthase
Source: eLife. 2026 Mar 13;14:RP109624. doi: 10.7554/eLife.109624 (PMC12987647; doi:10.7554/eLife.109624)
Supplement: Figure 4—figure supplement 2—source data 2. [file elife-109624-fig4-figsupp2-data2.pdf]

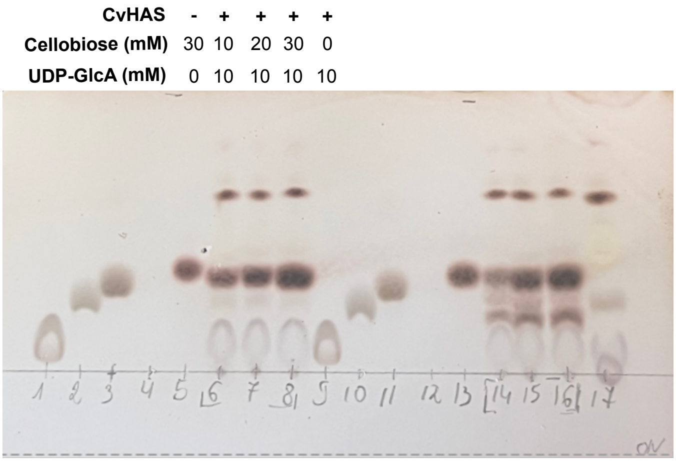

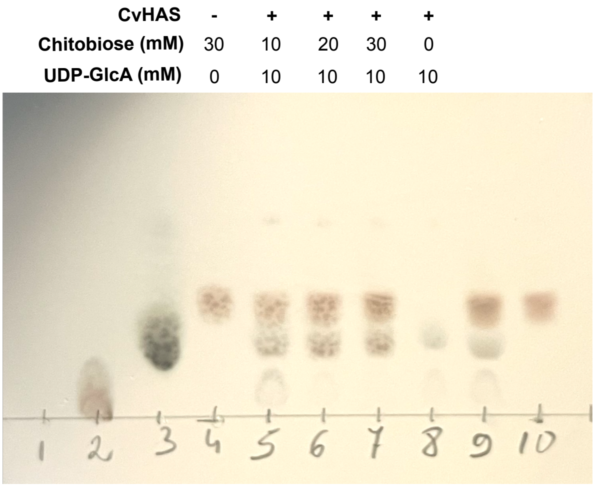

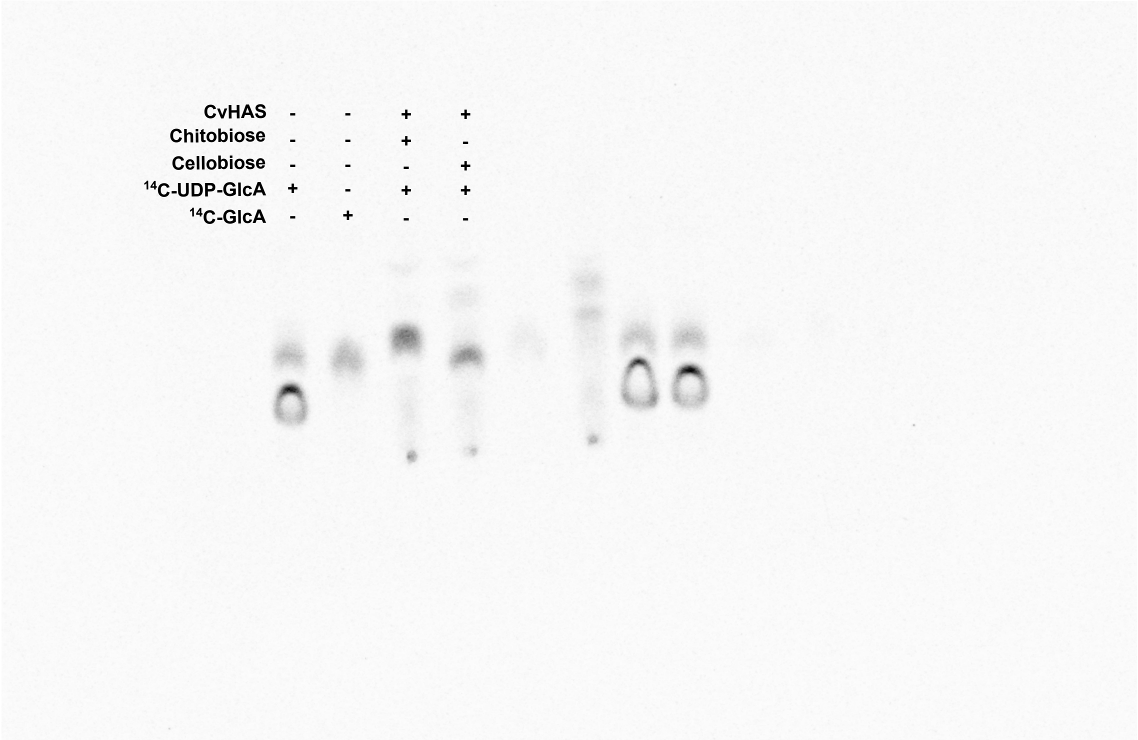

Figure 4 - figure supplement 2 - source data 2: Visualization of cellobiose and chitobiose glycosyl transfer products with UDP-GlcA generated by CvHAS using thymol, diphenylamine and 14C-GlcA based labeling
