# Supplementary material for: Insights into substrate binding and utilization by hyaluronan synthase
Source: eLife. 2026 Mar 13;14:RP109624. doi: 10.7554/eLife.109624 (PMC12987647; doi:10.7554/eLife.109624)
Supplement: Supplementary file 1. [file elife-109624-supp1.pdf]

	**UDP-GlcA, Inserted**<br>**EMDB ID:** EMD-73324<br>**PDB ID**: 9YQ5	**UDP-GlcA, Proofreading**<br>**EMDB ID:** EMD-73323<br>**PDB ID:** 9YQ4	**DDM Bound**<br>**EMDB ID:** EMD-73321<br>**PDB ID:** 9YQ2
**Data collection and processing**			
Magnification	81,000X	81,000X	130,000X
Voltage (kV)	300	300	300
Electron exposure (e- per Å^2)	50	50	60
Defocus range (μm)	-2.0 to -1.0	-2.0 to -1.0	-1.8 to -0.8
Pixel size (Å)	1.08	1.08	0.652
Symmetry imposed	C1	C1	C1
No. of initial particle images	6,567,299	6,567,299	6,386,906
No. of final particle images	110.407	64,146	513,368
Map resolution (Å)	2.8	3.3	2.9
FSC Threshold	0.143	0.143	0.143
Map resolution range (Å)	2.4 - 28.4	2.4 - 36.7	1.7 - 39.0
**Refinement**			
Initial model (PDB Code)	8snd	8snd	7sp9
Model resolution (Å)	2.8	3.3	2.9
FSC threshold	0.143	0.143	0.143
Map sharpening B factor (Å)	87.5	88.8	101.8
Model composition			
Nonhydrogen atoms	6034	5993	5939
Protein residues	742	739	731
Ligands	4	3	4
B-factors (Å^2)			
Protein	93.31	45.31	55.13
Ligands	110.36	46.85	74.2
RMSD			
Bond Lengths	0.002	0.015	0.002
Bond angles	0.432	0.985	0.466
Validation			
MolProbity score	1.57	1.95	1.69
Clashscore	5.36	8.34	7.29
Poor rotamers (%)	0.79	0.16	0.16
Ramachandran plot			
Favored (%)	95.91	91.66	95.84
Allowed (%)	4.09	8.34	4.16
Disallowed (%)	0	0	0
