# Supplementary material for: Insights into substrate binding and utilization by hyaluronan synthase
Source: eLife. 2026 Mar 13;14:RP109624. doi: 10.7554/eLife.109624 (PMC12987647; doi:10.7554/eLife.109624)
Supplement: Supplementary file 2. [file elife-109624-supp2.pdf]

DNA Primer List

T7_Term_Fwd	GAGCAATAACTAGCATAACCCCTTGGG
T7_Term_Rev	CCCAAGGGGTTATGCTAGTTATTGCTC
T7_Promoter_Fwd	CCCGCGAAATTAATACGACTCACTATAGG
T7_Promoter_Rev	CCTATAGTGAGTCGTATTAATTTCGCGGG
Y91F_QC_Fwd	CAGTCATAATCGCCGGCTTTCGCGAGGACCCTTTTATG
Y91F_QC_Rev	CATAAAAGGGTCCTCGCGAAAGCCGGCGATTATGACTG
Y91A_QC_Fwd	CAGTCATAATCGCCGGCGCGCGCGAGGACCCTTTTATG
Y91A_QC_Rev	CATAAAAGGGTCCTCGCGCGCGCCGGCGATTATGACTG
H174A_QC_Fwd	CATTTGTATCCTCCAGCCCGCGCGTGGTAAGCGGGAGAGTC
H174A_QC_Rev	GACTCTCCCGCTTACCACGCGCGGGCTGGAGGATACAAATG
H174W_QC_Fwd	CATTTGTATCCTCCAGCCCTGGCGTGGTAAGCGGGAGAGTC
H174W_QC_Rev	GACTCTCCCGCTTACCACGCCAGGGCTGGAGGATACAAATG
